# Targeting miR-18a sensitizes chondrocytes to anticytokine therapy to prevent osteoarthritis progression

**DOI:** 10.1038/s41419-020-03155-9

**Published:** 2020-11-03

**Authors:** Chengjie Lian, Tianyu Tao, Peiqiang Su, Zhiheng Liao, Xudong Wang, Yiting Lei, Pei Zhao, Lei Liu

**Affiliations:** 1 Department of Orthopedic Surgery, The First Affiliated Hospital of Chongqing Medical University, Chongqing, China; 2 Department of Orthopaedic Surgery, First Affiliated Hospital, Sun Yat-sen University, Guangzhou, Guangdong China; 3 Guangdong Provincial Key Laboratory of Orthopedics and Traumatology, First Affiliated Hospital, Sun Yat-sen University, Guangzhou, Guangdong China; 4 Department of Microbiology, Zhongshan School of Medicine, Sun Yat-Sen University, Guangzhou, Guangdong China; 5 Chongqing Key Laboratory of Molecular Oncology and Epigenetics, The First Affiliated Hospital of Chongqing Medical University, Chongqing, China

**Keywords:** Cell signalling, Pathogenesis

## Abstract

Inflammation participates in the development of OA and targeting inflammatory signaling pathways is a potential strategy for OA treatment. IL-1β is one of the most important inflammatory factors to trigger the activation of NF-κB signaling and accelerate OA progression, whereas OA patients could hardly benefit from inhibiting IL-1β in clinic, suggesting the importance to further explore the details of OA inflammation. We here showed that expression of miR-18a in chondrocytes was specifically induced in response to IL-1β in vitro as well as in rat model of OA during which NF-κB signaling was involved, and that nuclear-translocated p65 directly upregulated miR-18a expression at transcriptional level. Further, increased miR-18a mediated hypertrophy of chondrocytes, resulting in OA degeneration, by targeting TGFβ1, SMAD2, and SMAD3 and subsequently leading to repression of TGF-β signaling. And the level of serum miR-18a was positively correlated to severity of OA. Interestingly, other than IL-1β, pro-inflammation cytokines involving TNFα could also remarkably upregulate miR-18a via activating NF-κB signaling and subsequently induce chondrocytes hypertrophy, suggesting a pivotal central role of miR-18a in inflammatory OA progression. Thus, our study revealed a novel convergence of NF-κB and TGF-β signaling mediated by miR-18a, and a novel mechanism underlying inflammation-regulated OA dependent of NF-κB/miR-18a/TGF-β axis. Notably, in vivo assay showed that targeting miR-18a sensitized OA chondrocytes to IL-1β inhibitor as targeting IL-1β and miR-18a simultaneously had much stronger inhibitory effects on OA progression than suppressing IL-1β alone. Therefore, the diagnostic and therapeutic potentials of miR-18a for OA were also revealed.

## Introduction

Though OA has been considered as a wear and tear disease for long time, increasing evidence suggests that chronic low-grade inflammation plays a pivotal role in the onset and progress of OA^1^. Inflammatory mediators like IL-1β are synthesized, in part, in response to tissue injury and abnormal mechanical stress in the OA joints^2–4^. And they can induce hypertrophy, metabolic imbalance, dysregulation of autophagy, and apoptosis of chondrocytes, resulting in the degeneration of articular cartilage^5–10^. Among multiple pathological changes of chondrocytes caused by inflammation, pathological hypertrophy could disturb the homeostasis of both chondrocytes and cartilage matrix, and is thought to play a central role in OA development^11^. However, the detailed mechanisms by which OA inflammation caused chondrocyte hypertrophy remained largely unknown^11^. Besides, since some evidence showed that deletion of *Il1b* accelerated the development of OA lesions in the mouse model, cytokine played a complex role in maintaining cartilage homeostasis^12^, which needed further investigation.

The detailed mechanism underlying inflammatory microenvironment-mediated TGF-β signaling suppression and hypertrophy of chondrocytes in OA was not understood clearly. During OA development, several molecules have been reported to be involved to mediate TGF-β signaling suppression at a transcriptional level, EZH2 and CaMKII for instance^13,14^. However, inconsistency of SMAD2/3 protein and mRNA level was observed in OA as SMAD2/3 protein was down-regulated along with no alteration at mRNA level^11,15,16^ (GSE117999 and GSE110606), which could not be explained by the aforementioned mechanism, and suggested a post-translational regulation, such as microRNAs (miRNAs). miRNAs are a class of endogenous ~22 nt non-coding small RNAs of targeting mRNAs of multiple genes. They bind the 3′-untranslated region (3′UTR) of target mRNAs perfectly or imperfectly, resulting in post-transcriptional inhibition or mRNA cleavage and consequent reduced levels of their corresponding protein products. miRNAs are widely involved in modulation of various important biological processes in OA. For instance, miR-204/-211 loss-of-function induces matrix-degrading proteases in articular chondrocytes (ACs) and synoviocytes, thus stimulating articular cartilage destruction^17^. However, whether and how miRNAs participate in inflammation-mediated TGF-β signaling suppression and hypertrophy of chondrocytes remain to be investigated.

Current therapies for OA, such as analgesics, NSAIDs, and hyaluronic acid, only control the symptoms, and none of the FDA-approved treatments can prevent or slow the disease progression^18^. Safe and effective disease-modifying OA drugs (DMOADs) are urgently needed. Considering its central role in the initiation and progress of OA, inhibition of OA inflammation seems to be able to form the basis of such DMOADs. Several anti-inflammatory therapeutics, which targeting the key mediators like IL-1β, TNF, COX1, COX2, MMPs, and iNOS, have been tested in human OA, but till now the results are disappointing^1,19–24^. Since OA is a heterogeneous disease and a large number of inflammatory mediators are involved, targeting a single molecular could not inhibit OA effectively. Revealing the common downstream signaling pathway and the key molecular which mediate the OA inflammation was essential for developing novel strategy for OA treatment.

## Result

### miR-18a was up-regulated in response to IL-1β in OA

**Fig. 1:**
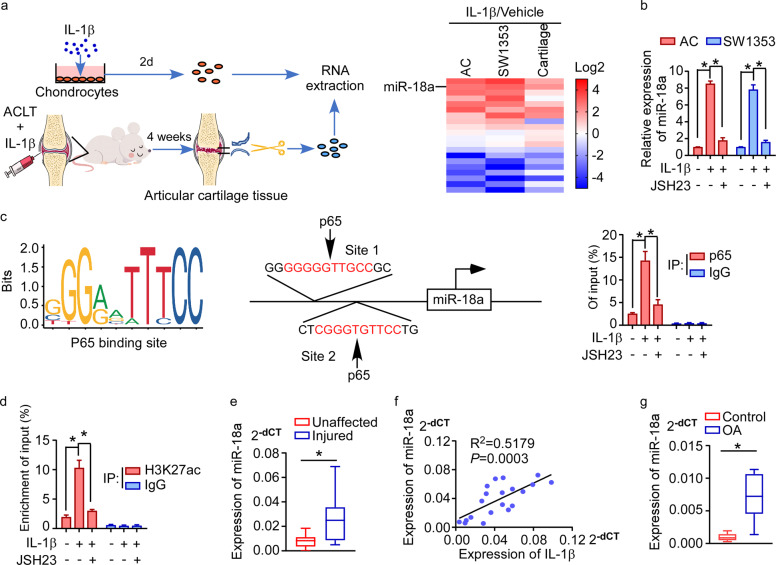
miR-18a was up-regulated in response to IL-1β in OA. **a** Schematic representation for identification of significantly altered miRNAs in stimulation of IL-1β in chondrocytes in vitro and in vivo simultaneously. In vitro, RNA was extracted from human articular chondrocytes and SW1353 cells cultured with IL-1β (10 ng/mL) for 2 days. ACLT operation was used to induce knee OA in rats, and the day after surgery vehicle or IL-1β (20 ng/joint) were injected into the rats’ knee joints every 3 days, followed by dissociation of articular cartilage after 4 weeks and extraction of its RNA. qRT-PCR shows the relative expression of miRNAs those had been found to be regulated by IL-1β in rat chondrocytes. **b** The effect of suppressing NF-κB signaling on the ability of IL-1β to induce expression of miR-18a. **c** Schematic diagram of potential binding site for p65 in the promoter region of miR-18a (left panel). ChIP enrichment assay shows binding of p65 to the predicted binding site in the promoter region of miR-18a in stimulation of IL-1β, which could be remarkably repressed by JSH23 (right panel). IgG immunoprecipitation was used as a negative control. **d** ChIP analysis following H3K27ac immunoprecipitation shows the interaction between H3K27ac and the promoter region of miR-18a in response to IL-1β with or without JSH23. IgG immunoprecipitation was used as a negative control. **e** qRT-PCR to assess the expression of miR-18a in injured articular cartilage of OA patients and the corresponding unaffected cartilage tissues. **f** Correlation between expression of miR-18a and IL-1β in OA lesions was determined by qRT-PCR and pearson correlation analysis. **g** Serum miR-18a was assessed in OA patients and healthy volunteer donors. Data in **b**–**e** and **g** are presented as mean ± SD. *P < 0.05.

To reveal the role of non-coding RNA in inflammation-regulated OA, we first screened miRNAs which were significantly differentially expressed in response to IL-1β, one of the most important inflammatory cytokines in OA microenvironment. As seen in Fig. 1a and Supplementary Table 1, expression of a series of miRNAs, which had been found to be regulated by IL-1β in rat chondrocytes (GSE33310), was assessed in stimulation of IL-1β both in vitro and in vivo. Notably, miR-18a was remarkably increased both in cartilage tissues from rat knee OA model with intra-articular injection of IL-1β and in human ACs and SW1353 cells (a human chondrosarcoma cell line) treated with IL-1β in vitro. As NF-κB signaling was the most important downstream pathway in inflammation cascades, and it also took part in the regulation of OA development, we asked if IL-1β induce expression of miR-18a in an NF-κB signaling-dependent way. We found that JSH-23, a compound effectively suppressing NF-κB signaling activity by inhibiting nuclear-translocation of p65, could abrogate IL-1β-induced up-regulation of miR-18a in ACs and SW1353 cells (Fig. 1b). Further, potential binding sites for p65 were found by analyzing the promoter sequences of miR-18a using the ECR browser and JASPAR database, which was conferred by CHIP assay (Fig. 1c). Supportively, these sites were also identified at an ENCODE H3K27ac site in the gene body, and the transcriptional activity was increased by IL-1β, which could be abrogated by JSH-23 (Fig. 1d). Thus, we speculated that IL-1β mediates expression of miR-18a by activating NF-κB signaling in OA chondrocytes. Moreover, we identified up-regulation of miR-18a in injured lesion of OA patients compared to that in paired unaffected cartilage and a positive correlation between the expression of miR-18a and IL-1β in OA injured cartilage, further supporting that miR-18a might participate in inflammation network-regulated progression of OA in clinic (Fig. 1e, f). Interestingly, we also observed remarkably higher level of miR-18a in the serum of OA patients compared to that in the serum of healthy controls (Fig. 1g), suggesting that serum miR-18a might be a potential biomarker for indicating inflammation subtype of OA.

### IL-1β induced-miR-18a accelerates OA progression by promoting chondrocyte hypertrophy

**Fig. 2:**
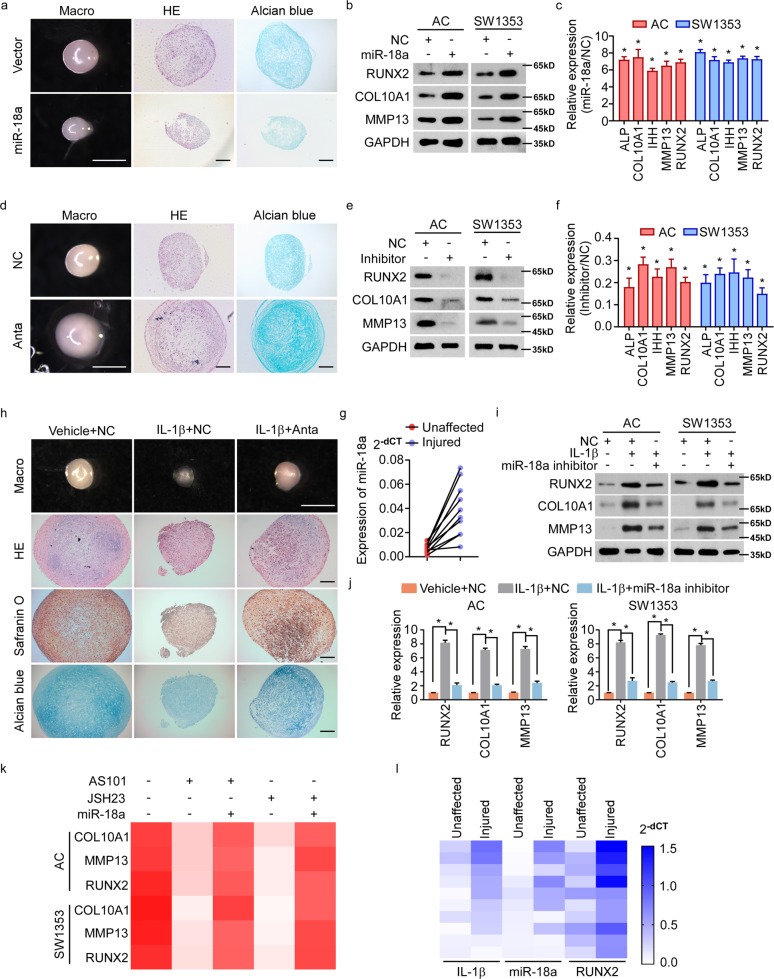
IL-1β induced-miR-18a accelerates OA progression by promoting chondrocyte hypertrophy. **a** Pellet cultures of MSCs expressing vector or miR-18a were induced to undergo chondrogenesis for 14 days and then induced for hypertrophic differentiation for another 14 days. Gross appearance, HE staining, and Alcian blue staining were evaluated. **b, c** WB **b** and qRT-PCR **c** to assess the expression of chondrocyte hypertrophy-related genes when miR-18a was ectopic expressed. **d** The effect of antagomir of miR-18a (Anta) on hypertrophy of chondrifying MSCs was assessed. **e**, **f** Expression of chondrocyte hypertrophy-related genes were determined by WB **e** and qRT-PCR **f** in AC and SW1353 cells transfected with inhibitor of miR-18a. **g** qRT-PCR shows miR-18a level in both injured cartilage and the corresponding paired unaffected cartilage. **h** The effect of miR-18a antagomir on IL-1β-enhanced hypertrophy of chondrifying MSCs was assessed. **i**, **j** Protein **i** and mRNA **j** levels of hypertrophy-related genes were analyzed in chondrocytes in response to IL-1β with or without inhibition of miR-18a. **k** qRT-PCR shows the effect of miR-18a ectopic expression on chondrocyte hypertrophy in cells treated with either AS101 or JSH23. **l** Expression of IL-1β, miR-18a, and RUNX2 were analyzed in injured cartilage and corresponding unaffected cartilage from OA patients. Scale bar in images of gross appearance in **a**, **d**, and **h**: 1 mm; other scale bars: 200 μm. Data in **c**, **f**, and **j** are presented as mean ± SD. *P < 0.05.

We next sought to investigate whether and how inflammation-induced miR-18a mediate development of OA. Chondrocyte hypertrophy was one of the pathological changes of chondrocytes caused by inflammation, which played a central role in OA initiation and progression. The effect of miR-18a on the hypertrophy of chondrifying mesenchymal stem cells (MSCs) was determined. Pellet cultures of human MSCs, those had been pre-infected with lentivirus encoding vector or miR-18a to generate vector or miR-18a-stably expressed MSCs (Fig. S1a), were induced to undergo chondrogenesis for 14 days and then induced to undergo hypertrophic differentiation for another 14 days. As seen in Fig. 2a, MSCs expressing miR-18a formed much smaller cartilage pellets than those expressing vector. And miR-18a could result in a less homogeneous hyaline cartilage-like morphology, with more hypertrophic chondrocytes. Similarly, ACs and SW1353 cells expressed more hypertrophy-related genes at both mRNA and protein levels when miR-18a was ectopically expressed (Figs. 2b, c and S1b). In contrast, cartilage pellets formed by MSCs with suppression of miR-18a were much larger than those formed by control MSCs. In parallel, more homogeneous hyaline cartilage-like morphology and less hypertrophic chondrocytes were observed when MSCs were treated with antagomir of miR-18a (Fig. 2d). Meanwhile, down-regulated hypertrophic markers could be seen in ACs and SW1353 cells with inhibition of miR-18a (Fig. 2e, f). In support, we identified significantly increased miR-18a in injured cartilages compared to their paired unaffected cartilages in OA patients (Fig. 2g), together with previous findings suggesting the critical role of miR-18a in chondrocyte hypertrophy and accelerating OA progression.

IL-1β-regulated inflammation network was reported to promote OA progression by inducing apoptosis, MMPs production, hypertrophy, etc., during which we wondered if IL-1β-induced miR-18a is involved. Figure 2h exhibited that inhibiting miR-18a could abrogate the facilitating effects of IL-1β on hypertrophy of chondrifying MSCs. And inhibition of miR-18a canceled the IL-1β-mediated increase of hypertrophy-related genes (Fig. 2i, j). Supportively, restored expression of miR-18a could reverse inhibiting IL-1β or NF-κB signaling-mediated suppression of hypertrophy, as re-expression of hypertrophy-related genes was observed when miR-18a was ectopic expressed in cells with treatment of AS101 (a compound inhibiting IL-1β converting enzyme) or JSH-23 (Fig. 2k). Besides, much higher level of miR-18a along with RUNX2 was seen in OA-injured cartilage tissues with much more expression of IL-1β (Fig. 2l). Taken together, miR-18a was supposed to play a critical role in IL-1β-activated NF-κB signaling-mediated hypertrophy and degeneration of OA chondrocytes.

### miR-18a suppresses TGF-β signaling by targeting TGFβ1, SMAD2, and SMAD3

**Fig. 3:**
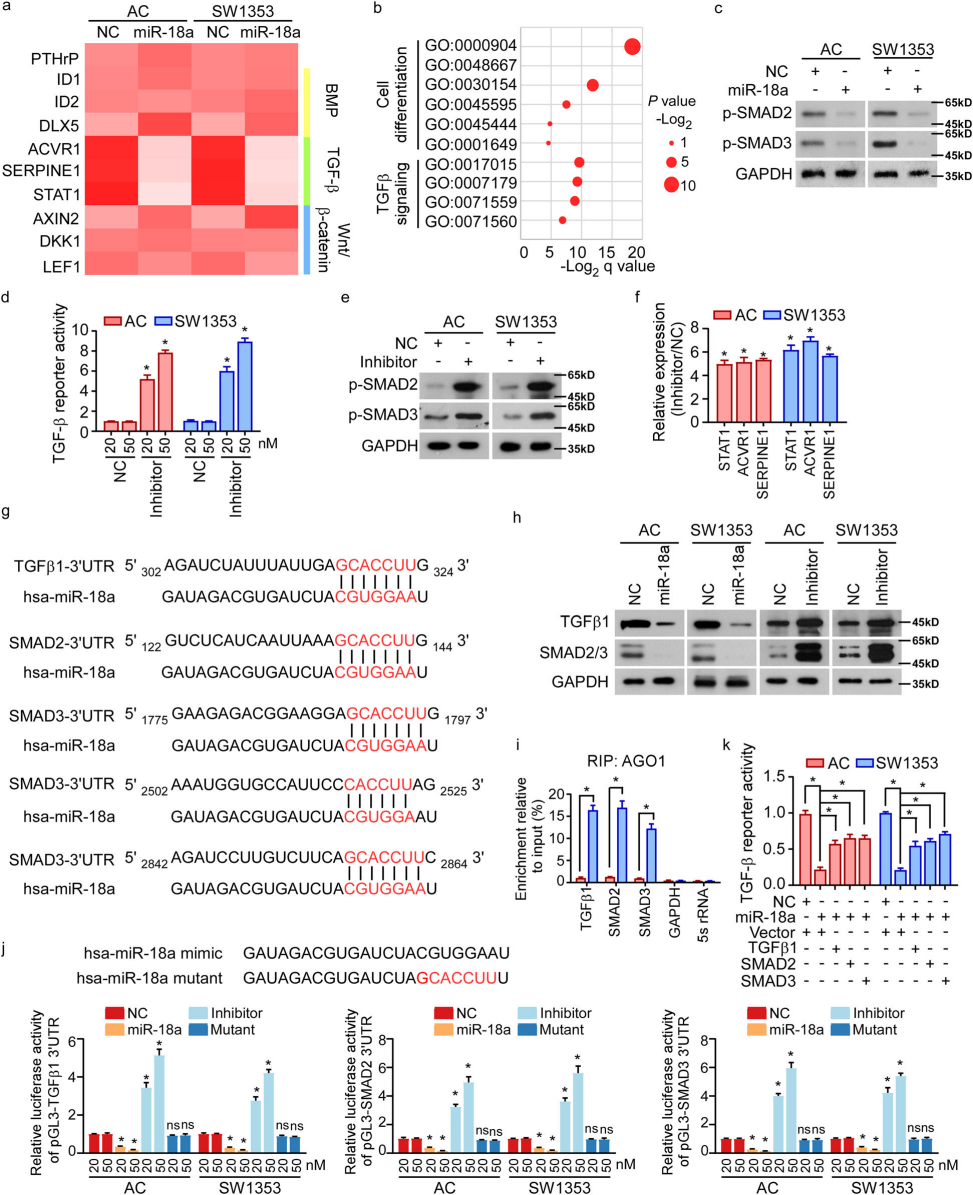
miR-18a suppresses TGF-β signaling by targeting TGFβ1, SMAD2, and SMAD3. **a** Expression of PTHrP and downstream genes of BMP, TGF-β, and Wnt/β-catenin signaling pathways was analyzed in miR-18a-overexpressed cells. **b** mRNA array was conducted in human ACs expressing control or miR-18a. And GO-enrichment analysis shows the correlation between expression of miR-18a and cell differentiation-associated signature or TGF-β signaling. **c** WB shows phosphorylation of SMAD2 and SMAD3 when miR-18a was overexpressed. **d** Dual-luciferase assays reveal TGF-β signaling activities. **e**, **f** Effect of inhibiting miR-18a on activity of TGF-β signaling was analyzed by WB **e** and qRT-PCR **f**. **g** Targetscan tool showing schematic representation of putative binding sites for miR-18a in 3′-UTRs of TGFβ1, SMAD2, and SMAD3. **h** WB analysis of the protein levels of TGFβ1 and SMAD2/3 in the indicated cells. **i** By immunoprecipitation against Ago1, RIP analysis reveals the interaction of miR-18a with the 3′-UTRs of TGFβ1, SMAD2, or SMAD3 mRNA to form miRNP complexes. IgG immunoprecipitation, as well as the interaction of miR-18a with GAPDH and 5s rRNA, were used as negative controls. **j** Luciferase assay of pGL3-TGFβ1-3′-UTR, pGL3-SMAD2-3′-UTR, and pGL3-SMAD3-3′-UTR reporters in the indicated cells, co-transfected with increasing amounts (20 and 50 nM) of the indicated oligonucleotides. The sequence of the miR-18a mutant is shown. **k** Effects of restored expression of TGFβ1, SMAD2, or SMAD3 in miR-18a-overexpressing cells on luciferase activities of the TGF-β reporter. Data in **d**, **f**, **i**, **j**, and **k** are presented as mean ± SD. *P < 0.05.

Hypertrophic differentiation of OA chondrocytes was complicated pathological processes involving various signaling pathways, including PTHrP, Wnt/β-catenin, BMP, and TGF-β signaling. We further investigated the detailed mechanism underlying miR-18a-mediated hypertrophy of chondrocytes. Among the chondrocyte hypertrophy-associated signaling pathways, we found that TGF-β signaling was remarkably repressed by miR-18a (Fig. 3a). In accordance with that, mRNA array and GO-enrichment analysis in human ACs with or without ectopic expression of miR-18a showed that miR-18a was correlated to signatures of cell differentiation and TGF-β signaling (Fig. 3b). We verified the inhibitory effects of miR-18a on TGF-β signaling by assessing the phosphorylation of SMAD2 and SMAD3 (Fig. 3c). In parallel, inhibitor of miR-18a could dose-dependently increase reporter activity of TGF-β signaling and phosphorylation of SMAD2 and SMAD3, accompanied by up-regulation of TGF-β signaling downstream genes (Fig. 3d–f).

Three positive regulators of the TGF-β pathway, TGFβ1, SMAD2, and SMAD3, predictively contain putative binding sites for miR-18a in their 3′-untranslated regions (UTRs; Fig. 3g). Protein levels, rather than mRNA levels, of these three genes were remarkably reduced in ACs and SW1353 cells overexpressing miR-18a but were upregulated in the miR-18a-inhibited cells (Figs. 3h and S2). RNA-immunoprecipitation analysis showed that the transcripts of these three candidate target genes were indeed specifically assembled into the miR-18a mimic oligonucleotides-containing miRNPs (Fig. 3i). Moreover, luciferase activity of the TGF-β signaling reporter linked with the 3′-UTR of each target gene was dose-dependently repressed by miR-18a mimic transfection, but reversely increased following miR-18a inhibition and mutation of miR-18a mimic completely abolished the repressive effects (Fig. 3j). Furthermore, re-expressing TGFβ1, SMAD2, and SMAD3 activated TGF-β pathway in miR-18a-overexpressing cells (Fig. 3k). Thus, we supposed that miR-18a inhibited TGF-β signaling by targeting 3′-UTR of TGFβ1, SMAD2, and SMAD3.

### miR-18a mediates chondrocyte hypertrophy via inhibiting TGF-β signaling

**Fig. 4:**
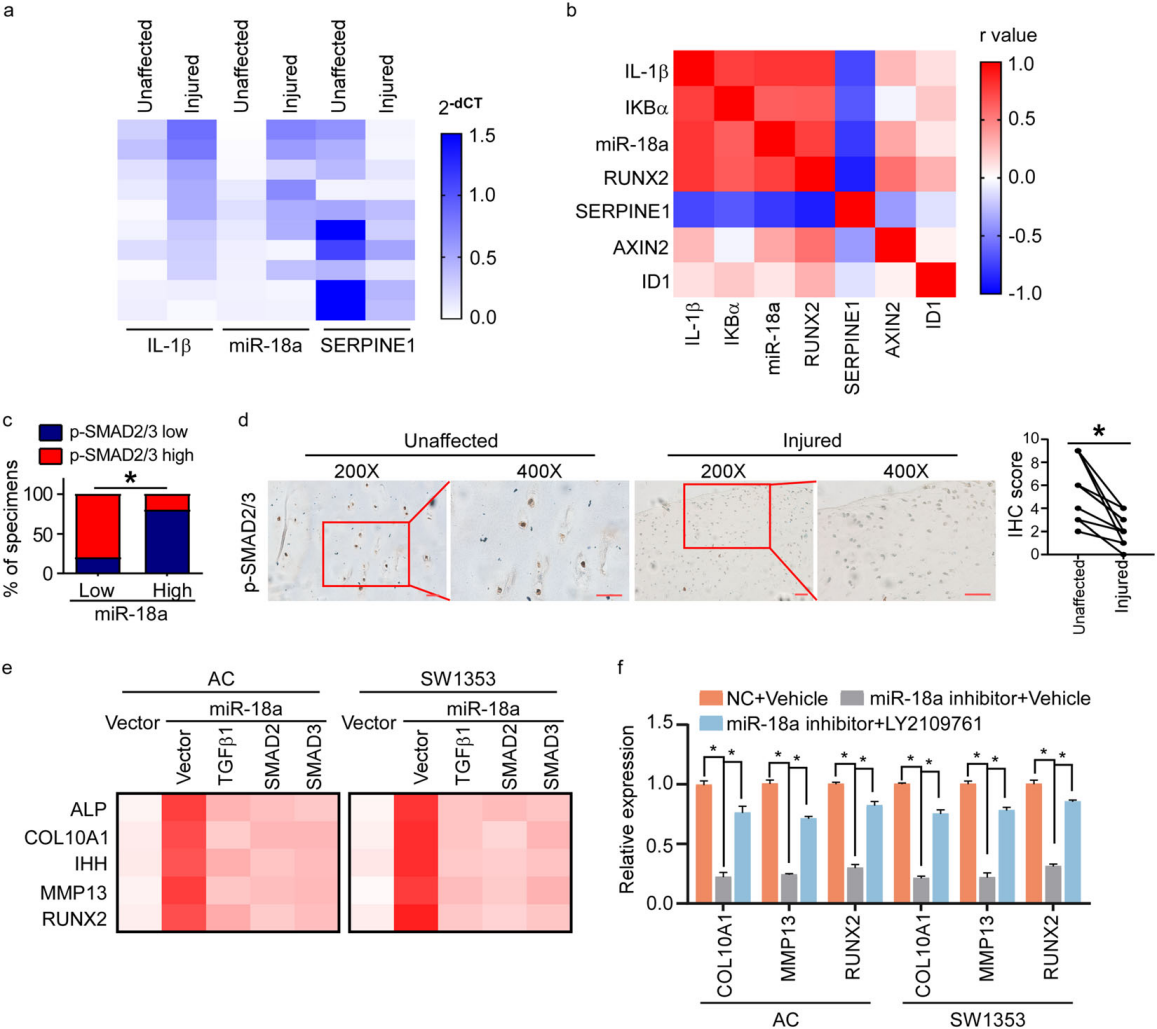
miR-18a mediates chondrocyte hypertrophy via inhibiting TGF-β signaling. **a** qRT-PCR to determine the expression of IL-1β, miR-18a, and SERPINE1 in injured cartilage and corresponding paired unaffected cartilage of OA patients. **b** Pearson’s correlation coefficient between IL-1β, IKBα (NF-κB signaling downstream gene), miR-18a, RUNX2, SERPINE1, AXIN2, and ID1 in injured lesions of OA patients was performed, and the heat map shows *r* values. **c** Correlation between p-SMAD2/3 and expression of miR-18a in OA lesions was assessed by IHC, qRT-PCR, and chi-square test. **d** IHC to determine the level of phosphorylation of SMAD2/3 in unaffected and injured articular cartilage. Scale bar: 50 μm. **e** Effects of restored expression of TGFβ1, SMAD2, or SMAD3 in miR-18a-overexpressing cells on expression of hypertrophy-related genes. **f** qRT-PCR exhibits the expression of hypertrophy-related genes in AC and SW1353 cells expressing inhibitor of miR-18a with or without LY2109761. Data in **f** are presented as mean ± SD. *P < 0.05.

The clinical relevance of miR-18a level with suppression of TGF-β signaling was analyzed. As seen in Fig. 4a, expression of SERPINE1 was much lower in OA injured cartilage tissues with high level of IL-1β and miR-18a than that in unaffected control cartilage tissues with low level of IL-1β and miR-18a. By analyzing the expression of IL-1β, IKBα, miR-18a, RUNX2, SERPINE1, AXIN2, and ID1, positive correlation between IL-1β, miR-18a, NF-κB signaling, and hypertrophy-associated marker was exhibited, while negative correlation between TGF-β signaling and IL-1β, miR-18a, NF-κB signaling, or hypertrophy-associated marker could been seen (Fig. 4b). In support, a negative correlation between phosphorylation of SMAD2/3 and miR-18a was seen in OA cartilage tissues, and lower level of p-SMAD2/3 could be detected in OA injured tissues than that in corresponding unaffected tissues (Fig. 4c, d), further indicating that miR-18a mediated the onset of OA in a TGF-β signaling-dependent way.

We further verified the critical role of miR-18a-regulated TGF-β signaling in hypertrophy of chondrocytes. As seen in Fig. 4e, re-expressing TGFβ1, SMAD2, and SMAD3 suppressed overexpression of miR-18a-mediated hypertrophy of chondrocytes. And LY2109761, a compound inhibiting TGF-β signaling pathway by suppressing activation of TGF-β receptor type I/II, abrogated the suppression of hypertrophy of chondrocytes induced by interfering miR-18a (Fig. 4f). Taken together, we speculated that miR-18a accelerate OA progression via targeting TGFβ1, SMAD2, and SMAD3, and accelerating hypertrophy of chondrocytes subsequently.

### IL-1β-mediated hypertrophy depends on miR-18a-induced suppression of TGF-β signaling

**Fig. 5:**
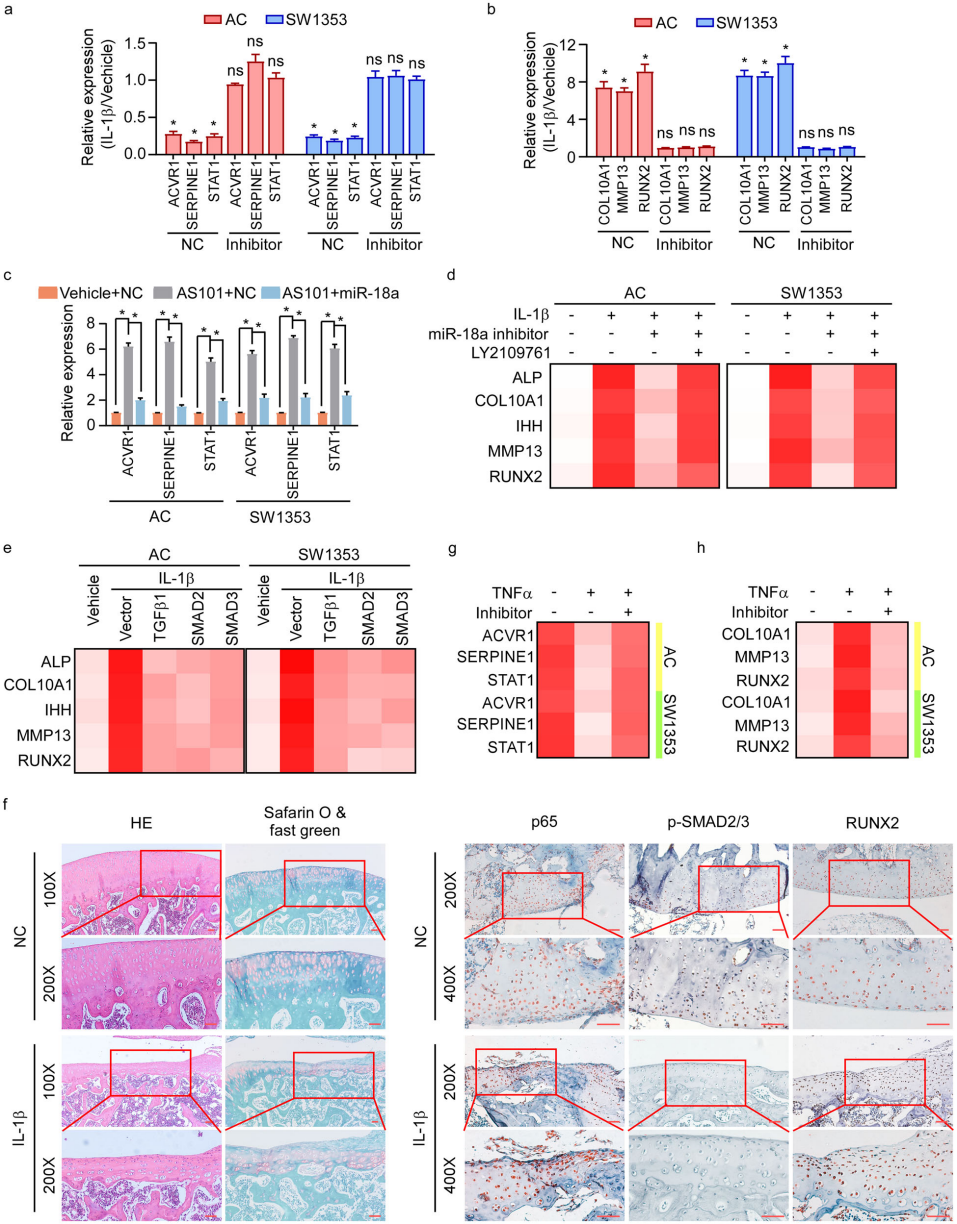
IL-1β-mediated hypertrophy depends on miR-18a-induced suppression of TGF-β signaling. **a** Effects of IL-1β on activation of TGF-β signaling in AC and SW1353 cells expressing NC or miR-18a inhibitor were assessed. **b** Expression of hypertrophy-related genes was analyzed in NC- or miR-18a inhibitor-expressed cells in response to IL-1β. **c** TGF-β signaling downstream genes expression was analyzed in IL-1β-suppressed cells with or without miR-18a re-expression. **d** Effects of LY2109761 on chondrocyte hypertrophy in cells with IL-1β treatment and miR-18a inhibitor expression were determined. **e** qRT-PCR shows the expression of hypertrophy-related genes when TGFβ1, SMAD2, or SMAD3 was restored expressed in cells with IL-1β stimulation. **f** Activity of NF-κB and TGF-β signaling, and expression of RUNX2 were determined in rat model of OA in Fig. 1a. Scale bar: 50 μm. **g**, **h** Activity of TGF-β signaling and the expression of hypertrophic genes in chondrocytes were assessed in cells in response to TNFα with or without inhibition of miR-18a. Data in **a**–**c** are presented as mean ± SD. *P < 0.05.

We further assessed the significance of miR-18a-TGF-β signaling axis in inflammation-mediated OA progression. Interestingly, IL-1β could induce suppression of TGF-β signaling in chondrocytes expressing NC, whereas that suppression could not be seen in cells with inhibition of miR-18a (Fig. 5a). In consistence, IL-1β could not mediate hypertrophy of chondrocytes in miR-18a-inhibited cells as it did in the corresponding control cells (Fig. 5b). And re-expression of miR-18a abrogated the down-regulation of TGF-β signaling activity mediated by IL-1β inhibitor (Fig. 5c). The results above indicated that IL-1β regulates TGF-β signaling and chondrocyte hypertrophy via miR-18a. Besides, the reversion on hypertrophy mediated by miR-18a inhibitor in cells with IL-1β treatment could be abrogated by LY2109761 (Fig. 5d). Restored expression of TGFβ1, SMAD2, or SMAD3 prevented the hypertrophy of chondrocytes mediated by IL-1β (Fig. 5e). Impressively, decreased p-SMAD2/3, increased activity of NF-κB signaling, and up-regulated RUNX2 were observed in rat model of knee OA with treatment of IL-1β (Fig. 5f). These findings elucidated a critical role of miR-18a-TGF-β signaling axis in IL-1β-regulated chondrocyte hypertrophy.

NF-κB signaling could be activated by a series of pro-inflammatory cytokines. We wondered whether other pro-inflammatory factors could also induce OA degeneration by inducing the expression of miR-18a. Impressively, TGF-β signaling could be suppressed by either TNFα, which was abrogated by inhibitor of miR-18a (Fig. 5g). And inhibiting miR-18a canceled the effects of TNFα on acceleration of chondrocyte hypertrophy (Fig. 5h). Our findings suggested that miR-18a might function as a critical mediator in inflammation-regulated chondrocyte hypertrophy in OA.

### Targeting miR-18a-sensitized chondrocytes in OA lesions to anticytokine drugs

**Fig. 6:**
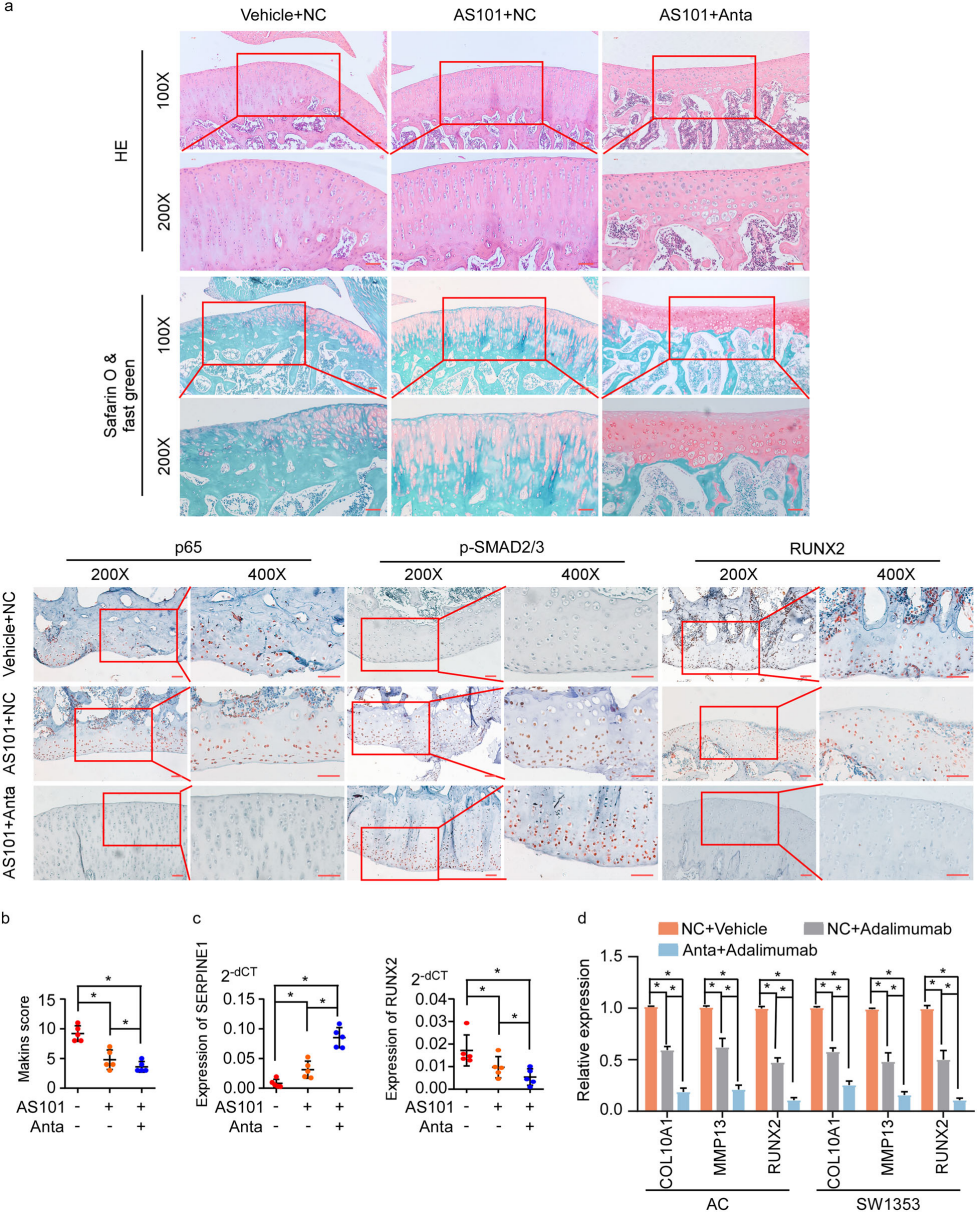
Targeting miR-18a sensitized chondrocytes in OA lesions to anticytokine drugs. **a** AS101 (1 mg/kg) was intraperitoneal injected, and 10 μL antagomir of miR-18a (250 nM) were intra-articular injected in rat model of knee OA every 3 days, followed by dissociation of knee joint after 4 weeks. HE staining, Safarin O, and fast green staining, and IHC of p65, p-SMAD2/3, and RUNX2 were conducted. Scale bar: 50 μm. **b** Mankin’s score was assessed. **c** Expression of SERPINE1 and RUNX2 in articular cartilage from OA rats with administration of AS101 or combination of AS101 and miR-18a antagomir. **d** qRT-PCR to determine the expression of hypertrophy-related genes in human ACs and SW1353 cells with treatment of adalimumab or combination of adalimumab and miR-18a antagomir. Data in **b**–**d** are presented as mean ± SD. *P < 0.05.

In clinic, targeting cytokine was supposed to be an approach for OA treatment, such as anti-IL-1β, whereas the therapeutic effect of IL-1β inhibitor was unsatisfactory. According to our findings previously, we speculated that various pro-inflammation factors induced NF-κB signaling activation and miR-18a expression might have contributed to the poor effect of IL-1β inhibitor. Notably, in our rat model of OA, combination of IL-1β inhibitor and antagomir of miR-18a had better effects in activating TGF-β signaling and suppressing hypertrophy of chondrocytes than IL-1β inhibitor, indicating that anti-miR-18a sensitized OA chondrocytes to IL-1β inhibitor (Fig. 6a–c). Supportively, miR-18a antagomir also increased sensitivity of OA chondrocytes to TNF-α antibody (adalimumab) as remarkably lower hypertrophic markers could be seen in cells with combination of miR-18a inhibitor and adalimumab (Fig. 6d), indicating that targeting miR-18a could increase the sensitivity of chondrocytes to cytokines drugs by inhibiting inflammation-regulated hypertrophy.

The findings aforementioned strongly suggested a novel mode of inflammation-regulated OA that inflammation-induced miR-18a-directed hypertrophy of chondrocytes contributed to OA progression through suppressing TGF-β signaling, and the potentials of targeting NF-κB/miR-18a/TGF-β axis in the treatment of OA (Fig. S3).

## Discussion

Inflammation plays an integral role in the progression of OA. Inflammatory cytokines, including IL-1β, TNFα, IL-6, and IL-8 secreted by the chondrocytes, synoviocytes, and infiltrating immune cells of OA joints are the main players^3,4^. However, phase I/II clinical trials testing the anticytokines drugs for OA patients usually ended up with failures because of poor therapeutic efficacies^19–25^. Thus, it is important to better understand the details of OA-associated inflammatory network in order to identify novel therapeutic strategies. In this study, NF-κB signaling, as the key common downstream pathway of pro-inflammatory factors, was found to be activated and directly up-regulate expression of miR-18a at transcriptional level. Induced miR-18a accelerated chondrocyte hypertrophy by suppressing TGF-β signaling, which finally conferred inflammation-mediated OA progression. Quite a lot of differentially expressed miRNAs have been identified in OA patients, and the broad biological significance of miRNAs on cartilage degeneration and OA progression has been investigated^26,27^. MiR-18a has been reported to target the 3′-UTR of CCN2, repress CCN2 expression, and significantly repress the mature chondrocytic phenotype, suggesting it to be a risk factor for cartilage degeneration. In this study, we confirmed its harmful role in OA development, and figured out the new target molecules and the mechanisms by which miR-18a promoted chondrocyte hypertrophy and cartilage degeneration^28^. Moreover, we revealed the key role of miR-18a in mediating the inflammation-induced OA progression and provided fruitful underlying targets to prevent inflammation-induced cartilage degeneration.

Till now, the effects of several drugs targeting inflammatory mediators have been studied on OA in human trials, however, the results are disappointing^19–25^. Since diverse proinflammatory mediators are involved in OA, targeting a single one seems to be insufficient. Uncovering and targeting the common downstream signaling pathway and the key molecular which mediate the OA inflammation might be a promising way to prevent cartilage degeneration caused by OA inflammation. Our study showed that NF-κB signaling-miR-18a axis, which could be activated by various inflammatory factors, played a central role in OA inflammation network. Besides, aiming to seeking for effective targets for OA therapy, we revealed the detailed mechanism underlying NF-κB signaling-miR-18a axis-driven OA and potentials to targeting this axis to prevent OA development. Thus, our findings not only uncovered a novel and central mechanism mediating inflammation-related OA, but also highlighted the clinical significance of NF-κB signaling-miR-18a axis in OA treatment.

Whether and how the convergence of NF-κB and TGF-β signaling participate in inflammation-mediated hypertrophy of chondrocyte in OA was not clearly understood. Previous reports showed that IL-1β and TNFα could inhibit TGF-β-induced SMAD2/3 transcriptional activity^29^. And down-regulation of IL-1β and TNFα induced by LBP was accompanied by up-regulation of TGFβ1^30^. Interestingly, regulation of NF-κB by TGF-β signaling was also observed. For instance, TGFβ1 stimulation increased expression of IL-1β and TNFα^31^. IKBα degradation was remarkably repressed in response to TGFβ1, leading to increased nuclear-located p65 subsequently^32^. Thus, a complex regulatory network involving NF-κB and TGF-β signaling was identified in OA inflammatory microenvironment. Aiming at the detailed regulatory mechanism of the crosstalk of NF-κB and TGF-β signaling, we found the inconsistency between mRNA and protein level of several key factors in TGF-β signaling pathway. Excitingly, we found that miR-18a, which was directly transcriptional up-regulated by NF-κB in response to inflammatory factors, IL-1β and TNFα, suppressed TGF-β signaling activity by targeting 3′-UTR of TGFβ1, SMAD2, and SMAD3, resulting in hypertrophy of chondrocytes. Results of our study revealed a novel mechanism of regulation of TGF-β by NF-κB at a post-transcriptional way. Besides, we proposed that miR-18a played a central role not only in IL-1β/TNFα-NF-κB-mediated suppression of TGF-β signaling, but also in inflammatory environment-mediated hypertrophy of chondrocytes and OA degeneration.

Previous findings suggested that OA patients might benefit from targeting IL-1β as inhibiting IL-1β signaling could repress the OA-associated inflammation and prevent chondrocyte hypertrophy in vitro and in animals^33–35^. However, a phase II clinical trial showed that patients with OA could hardly benefit from AMG108, IL-1 receptor antibody^22^, indicating that other than IL-1β, various inflammatory factors and signaling pathways which were involved in the regulation of complex inflammatory network contributed to OA progression. Notably, we noticed that miR-18a, which was directly up-regulated in response to IL-1β, promoted hypertrophy of chondrocytes by targeting TGF-β signaling. Importantly, increased serum miR-18a was detected and the level of serum miR-18a was positively correlated to the severity of OA. These findings strongly suggested that miR-18a was a potential target for treatment of OA as well as a biomarker for indicating inflammatory subtype of OA. Interestingly, as a critical convergence between NF-κB and TGF-β signaling, miR-18a could be induced by not only IL-1β, but also TNFα, via activating NF-κB signaling. The up-regulation of miR-18a in stimulation to various inflammatory factors might contribute to the failure of IL-1β receptor antibody in OA treatment. We next assessed whether anticytokine therapy along with inhibiting miR-18a could effectively repress inflammation-mediated development of OA and prevent cartilage destruction. Impressively, in rats, combination of IL-1β inhibitor and antagomir of miR-18a could remarkably reverse chondrocyte hypertrophy and OA progression by abrogating the repression of TGF-β signaling, indicating that targeting proinflammation cytokines and miR-18a simultaneously might represent an attractive strategy to counteract OA.

Among multiple pathological changes of chondrocytes caused by inflammation, hypertrophy plays a central role in OA cartilage degeneration. As reported by our previous study, chondrocyte hypertrophy could initiate and aggravate OA development through a disease amplifying loop^11^. Thus, not only inhibition of inflammation, but also restoration of the function and differentiation of chondrocytes, are necessary to protect articular cartilage from degeneration. Supportively, our findings that targeting IL-1β and miR-18a simultaneously, via which inflammation and chondrocyte hypertrophy were remarkably repressed, could effectively prevent OA progression as the disease amplifying loop identified before was blocked.

## Materials and methods

### Source of human cartilage and serum

Human cartilage samples were obtained from 20 OA patients (17 women and 3 men with a mean age of (68.30 ± 7.90) years) classified as grades 3 and 4 according to the Kellgren and Lawrence osteoarthritis grading system. The patients underwent total knee arthroplasty at the First Affiliated Hospital of Chongqing Medical University from 2019 to 2020. Cartilage samples were taken from both the injured area (mostly from the rim of the ulcer, comprising ~3–4 mm of the surrounding tissue) and the macroscopically unaffected area distal to the damaged zone. Measurements of mRNA and miRNA expressions and histology were performed. The cartilage samples were kept frozen at −80 °C until use or placed in paraformaldehyde for the histological study.

Human serum was taken from 10 OA patients mentioned above (7 women and 3 men with a mean age of (68.70 ± 7.63) years) and 10 age- and gender-matched non-OA healthy volunteer donors (7 women and 3 men with a mean age of (68.40 ± 6.83) years). Expression of miR-18a was examined.

Investigators were blinded to the different groups during data collection and subsequent data analysis.

### Induction and treatment of rat knee OA model

Male Sprague-Dawley rats (8–9 weeks of age, 260–280 g) were purchased and housed under a 12-hour light–dark cycle with free access to food and fresh water at room temperature. Five rats per group were used to ensure the adequate power, and rats with different weights were randomly allocated. Anterior cruciate ligament transection (ACLT) was used to induce knee OA in rat model as previously described^36^. The sample size of animal experiment was chosen based on the preliminary experiments, and no statistical methods were used. Investigators were blinded to the treatment groups during data collection and analysis.

To investigate the effects of IL-1β on the progression of OA, 20 ng IL-1β (R&D Systems) was injected by intra-articular every 3 days for 4 weeks from the day after ACLT operation. To investigate the effects of the combinations of AS101 with antagomir of miR-18a on OA progression, AS101 (1 mg/kg) (MedChemExpress, Monmouth Junction, NJ, USA) was injected by i.p. every 3 days for 4 weeks, and 10 μL antagomir of miR-18a (250 nM) (Ribobio, Guangzhou, China) were injected by intra-articular every 3 days for 4 weeks, from the day after ACLT operation. The rats were sacrificed at week 4 post-operatively. Samples of articular cartilage and subchondral bone of each knee were collected for further studies.

### Study approval

All experimental procedures involving animals met the relevant guidelines for the humane care of laboratory animals and were approved by the Institutional Animal Care and Use Committee of Chongqing Medical University. For human studies, prior patients’ consents and approval from the Institutional Research Ethics Committee of the First Affiliated Hospital of Chongqing Medical University were obtained.

More details about the materials and methods were listed in supplementary information.

## Supplementary information

Supplementary Figure Legends

Supplementary figure 1

Supplementary figure 2

Supplementary figure 3

Supplementary Table

Supplementary Information
