# Supplementary material for: Targeting miR-18a sensitizes chondrocytes to anticytokine therapy to prevent osteoarthritis progression
Source: Cell Death Dis. 2020 Nov 3;11(11):947. doi: 10.1038/s41419-020-03155-9 (PMC7609664; doi:10.1038/s41419-020-03155-9)
Supplement: Supplementary file 1 — Supplementary Figure Legends [file 41419_2020_3155_MOESM1_ESM.docx]

**Supplementary figure legends**

**Figure S1.** **a** MSCs stably overexpressing miR-18a were constructed as confirmed by qRT-PCR. **b** Expression of miR-18a was assessed in AC and SW1353 cells with ectopic expression of miR-18a.

**Figure S2.** mRNA level of TGF-β, SMAD2, and SMAD3 was determined by qRT-PCR in chondrocytes when miR-18a was ectopic expressed or suppressed.

**Figure S3.** Model for inflammation-induced miR-18a-mediated suppression of TGF-β signaling and acceleration of chondrocyte hypertrophy.
