# Supplementary figures and images for: Targeting miR-18a sensitizes chondrocytes to anticytokine therapy to prevent osteoarthritis progression
Source: Cell Death Dis. 2020 Nov 3;11(11):947. doi: 10.1038/s41419-020-03155-9 (PMC7609664; doi:10.1038/s41419-020-03155-9)

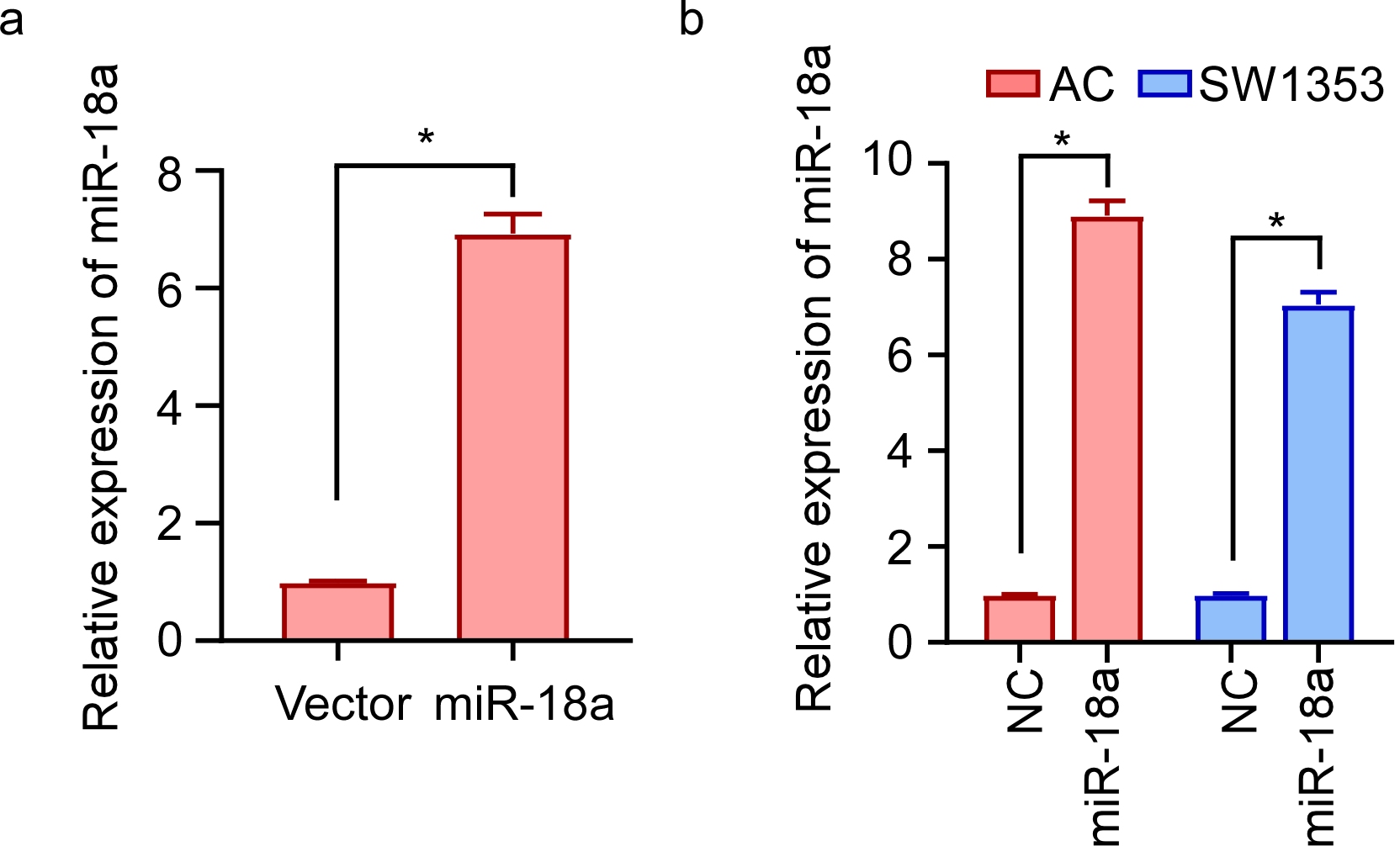

Supplement: Supplementary file 2 — Supplementary figure 1 [file 41419_2020_3155_MOESM2_ESM.tif]

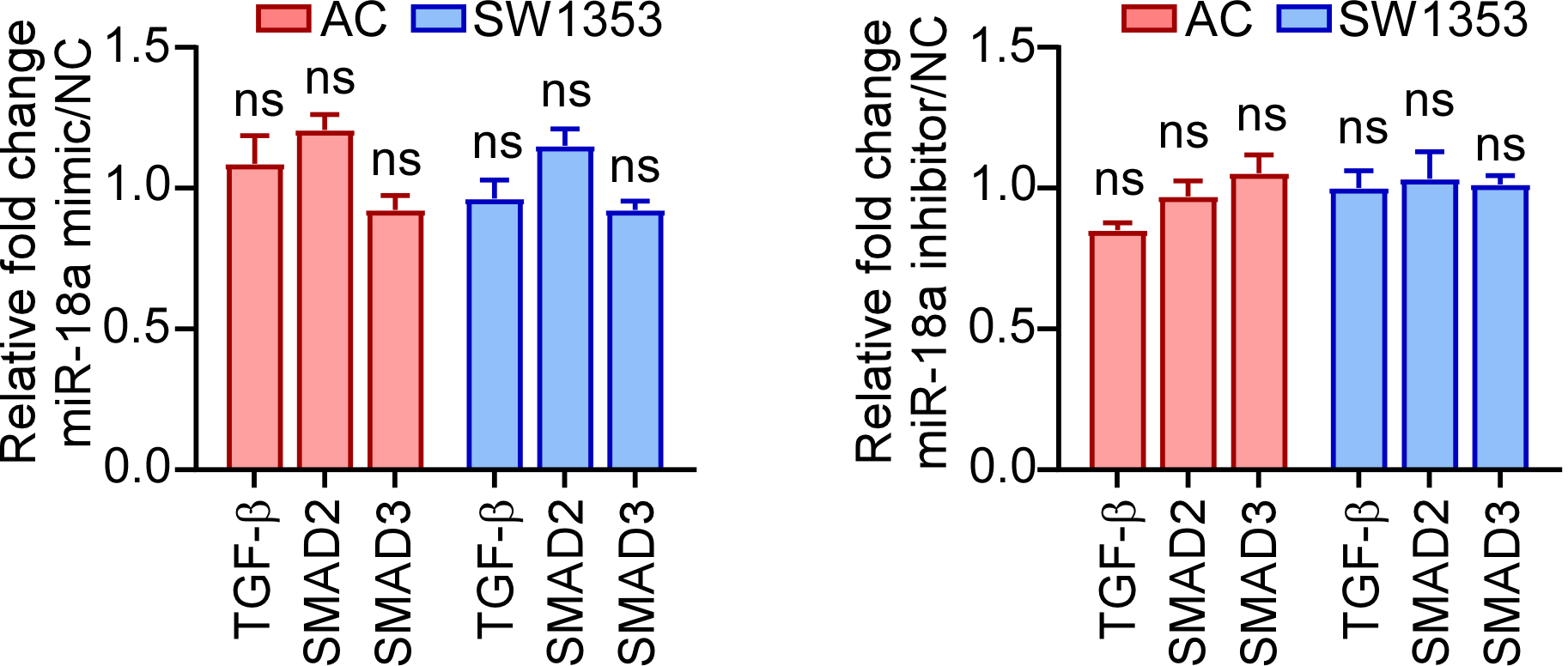

Supplement: Supplementary file 3 — Supplementary figure 2 [file 41419_2020_3155_MOESM3_ESM.tif]

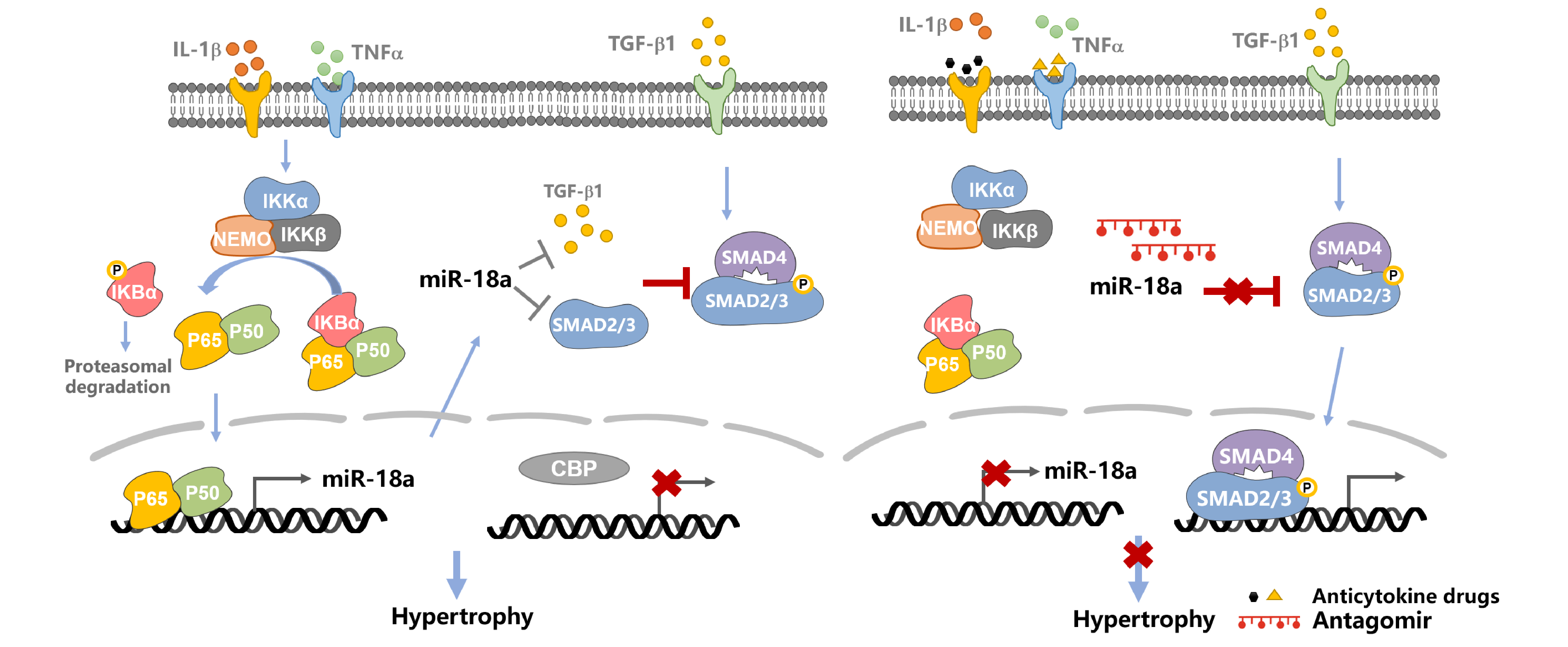

Supplement: Supplementary file 4 — Supplementary figure 3 [file 41419_2020_3155_MOESM4_ESM.tif]
