# Supplementary material for: Targeting miR-18a sensitizes chondrocytes to anticytokine therapy to prevent osteoarthritis progression
Source: Cell Death Dis. 2020 Nov 3;11(11):947. doi: 10.1038/s41419-020-03155-9 (PMC7609664; doi:10.1038/s41419-020-03155-9)
Supplement: Supplementary file 5 — Supplementary Table [file 41419_2020_3155_MOESM5_ESM.docx]

**Supplementary Table 1**

Relative fold change of miRNAs in cells in response to IL-1β

| miRNAs | Change Fold | | |
| --- | --- | --- | --- |
|  | AC | SW1353 | Cartilage |
| miR-29b-1 | 8.814 | 14.346 | 3.244 |
| miR-18a | 6.525 | 8.325 | 4.353 |
| miR-20b | 7.285 | 6.496 | 1.517 |
| miR-424 | 2.925 | 10.536 | 0.917 |
| miR-376B | 6.837 | 6.274 | 2.278 |
| miR-495 | 9.453 | 3.122 | 5.486 |
| miR-146a | 2.118 | 9.363 | 4.732 |
| miR-218 | 2.239 | 3.191 | 0.267 |
| miR-494 | 1.514 | 2.168 | 1.941 |
| miR-875 | 1.231 | 0.805 | 1.322 |
| miR-323 | 0.821 | 0.932 | 1.413 |
| miR-665 | 0.792 | 1.117 | 2.135 |
| miR-328 | 0.462 | 0.604 | 0.469 |
| miR-582 | 0.015 | 0.328 | 0.18 |
| miR-139 | 0.222 | 0.065 | 0.363 |
| miR-708 | 0.124 | 0.026 | 0.170 |
| miR-329-1 | 0.083 | 0.246 | 0.871 |
| miR-219-1 | 0.191 | 0.003 | 0.087 |
| miR-1249 | 0.056 | 0.154 | 0.793 |
| miR-33 | 0.186 | 0.002 | 0.285 |
