# Supplementary material for: Targeting miR-18a sensitizes chondrocytes to anticytokine therapy to prevent osteoarthritis progression
Source: Cell Death Dis. 2020 Nov 3;11(11):947. doi: 10.1038/s41419-020-03155-9 (PMC7609664; doi:10.1038/s41419-020-03155-9)
Supplement: Supplementary file 6 — Supplementary Information [file 41419_2020_3155_MOESM6_ESM.docx]

**Supplemental materials and methods**

**Cell culture**

The human chondrosarcoma cell lines SW1353 was purchased from American Type Culture Collection (Manassas, VA, USA). The 293T cell line was purchased from the China Center for Type Culture Collection (Wuhan, China). SW1353 cells were cultured in Dulbecco’s modified Eagle medium (DMEM)/F-12 medium (HyClone, Logan City, UT, USA) supplemented with 10% fetal bovine serum (FBS) (HyClone). 293T cells were cultured in DMEM medium (HyClone) supplemented with 10% FBS. Human articular chondrocytes were obtained from ScienCell (Carlsbad, CA, USA) and cultured in DMEM/F-12 supplemented with 10% FBS. Authenticity of the cell lines was verified by short tandem repeat (STR) fingerprinting at the Guangzhou Cellcook Biotech Co., Ltd. (Guangzhou, China). All cell lines were tested negative for mycoplasma contamination.

**Isolation and culture of human MSCs**

Human MSCs were isolated from bone marrow obtained from healthy volunteer donors as described previously (11, 36). Briefly, the bone marrow samples (8-10 mL) were diluted with 10 mL of phosphate-buffered saline (PBS). Cells were then fractionated on a lymphoprep density gradient by centrifugation at 500 g for 20 min. Interfacial mononuclear cells were collected, resuspended in low-glucose DMEM (HyClone) supplemented with 10% FBS, seeded and incubated at 37°C/5% CO_2_. After 48 h, nonadherent cells were removed by changing the medium. Thereafter, the medium was changed every 3 d. When the cells reached 80–90% confluence, they were trypsinized, counted, and plated again. Cells from passages 3–6 were used for the experiments.

**Chondrogenic** **and hypertrophic differentiation of human MSCs**

A high-density pellet culture system was applied for the chondrogenic and hypertrophic differentiation of human MSCs, as described previously (11). Cells were induced for chondrogenic pre-differentiation for 14 d in pellet culture (200,000 cells/pellet) in chondrogenic medium consisting of high-glucose DMEM with 50 μg/mL ascorbate acid 2-phosphate (Sigma-Aldrich, St. Louis, MO, USA), 40 μg/mL proline (Sigma-Aldrich), 10 ng/mL recombinant human TGFβ3 (R&D Systems, Minneapolis, MN, USA), and 1% ITS Universal Culture Supplement Premix (BD Biosciences, San Jose, CA, USA). To further induce hypertrophic differentiation, chondrogenic differentiated pellets were exposed to hypertrophic differentiation medium consisting of high-glucose DMEM with 50 μg/mL ascorbate acid 2-phosphate, 40 μg/mL proline, 1 nmol/L dexamethasone (Sigma-Aldrich), 1% ITS Universal Culture Supplement Premix, and 1 nmol/L triiodothyronine (Sigma-Aldrich) for 14 d. The medium was changed every 3 d.

**Real-time PCR**

Real-time PCR was performed on a Roche LightCycler 480 System (Roche, Basel, Switzerland) using SYBR Green Real-time PCR Master Mix (TOYOBO, Osaka, Japan). Each reaction was processed in triplicate, and an average ΔCt value from the whole group was taken. The relative expression levels of each gene were obtained using the 2^−ΔΔCt^ method.

Real-time PCR primers used are listed as the following:

Human

IL-1β-sense: 5’- ATGATGGCTTATTACAGTGGCAA -3’;

IL-1β-Antisense: 5’- GTCGGAGATTCGTAGCTGGA -3’;

ALP-Sense: 5’- GTGAACCGCAACTGGTACTC -3’;

ALP -Antisense: 5’- GAGCTGCGTAGCGATGTCC -3’;

COL10A1-Sense: 5’- ATGCTGCCACAAATACCCTTT -3’;

COL10A1-Antisense: 5’- GGTAGTGGGCCTTTTATGCCT -3’;

IHH-Sense: 5’- AACTCGCTGGCTATCTCGGT -3’;

IHH-Antisense: 5’- GCCCTCATAATGCAGGGACT -3’;

MMP13-Sense: 5’- ACTGAGAGGCTCCGAGAAATG -3’;

MMP13-Antisense: 5’- GAACCCCGCATCTTGGCTT -3’;

RUNX2-Sense: 5’- TGGTTACTGTCATGGCGGGTA -3’;

RUNX2-Antisense: 5’- TCTCAGATCGTTGAACCTTGCTA -3’;

ID1-Sense: 5’- CTGCTCTACGACATGAACGG -3’;

ID1-Antisense: 5’- GAAGGTCCCTGATGTAGTCGAT -3’;

ID2-Sense: 5’- AGTCCCGTGAGGTCCGTTAG -3’;

ID2-Antisense: 5’- AGTCGTTCATGTTGTATAGCAGG -3’;

DLX5-Sense: 5’- TTCCAAGCTCCGTTCCAGAC -3’;

DLX5-Antisense: 5’- GAATCGGTAGCTGAAGACTCG -3’;

ACVR1-Sense: 5’- GTGAAGGTCTCTCCTGCGGTA -3’;

ACVR1-Antisense: 5’- GCCATCGTTGATGCTCAGTGA -3’;

SERPINE1-Sense: 5’- ACCGCAACGTGGTTTTCTCA -3’;

SERPINE1-Antisense: 5’- TTGAATCCCATAGCTGCTTGAAT -3’;

STAT1-Sense: 5’- CAGCTTGACTCAAAATTCCTGGA -3’;

STAT1-Antisense: 5’- TGAAGATTACGCTTGCTTTTCCT -3’;

AXIN2-Sense: 5’- CAACACCAGGCGGAACGAA -3’;

AXIN2-Antisense: 5’- GCCCAATAAGGAGTGTAAGGACT -3’;

DKK1-Sense: 5’- CCTTGAACTCGGTTCTCAATTCC -3’;

DKK1-Antisense: 5’- CAATGGTCTGGTACTTATTCCCG -3’;

LEF1-Sense: 5’- AGAACACCCCGATGACGGA -3’;

LEF1-Antisense: 5’- GGCATCATTATGTACCCGGAAT -3’.

IKBα-Sense: 5’- CTCCGAGACTTTCGAGGAAATAC -3’;

IKBα-Antisense: 5’- GCCATTGTAGTTGGTAGCCTTCA -3’;

GAPDH-Sense: 5’- AGAAAAACCTGCCAAATATGATGAC -3’;

GAPDH-Antisense: 5’- TGGGTGTCGCTGTTGAAGTC -3’.

Rat

Runx2-Sense: 5’- TCCAACCCACGAATGCACTAC -3’;

Runx2-Antisense: 5’- GTAGTGAGTGGTGGCGGACT -3’;

Serpine1-Sense: 5’- GCTGATGGAGCCTTGAGA -3’;

Serpine1-Antisense: 5’- GGAAAGATTTACCAGTGCC -3’.

Gapdh-Sense: 5’- TGAACGGGAAGCTCACTGG -3’;

Gapdh-Antisense: 5’- TCCACCACCCTGTTGCTGTA -3’.

**Chromatin immunoprecipitation (ChIP).**

For ChIP analysis, 5×10^7^ SW1353 cells cultured in 10-cm culture dishes were harvested for cross-linking and sheared by sonication. The resultant chromatin fraction was immunoprecipitated using 10 μg antibodies against H3K27ac and p65 (Abcam, Cambridge, MA, USA) or negative control anti-IgG (Sigma-Aldrich). After reversing the cross-links with NaCl and removing proteins with proteinase K, enriched DNA fragments were purified and isolated via phenol/chloroform extraction and ethanol precipitation. The final DNA pellets were then subjected to real-time quantitative PCR with the indicated specific primers (1).

**RNA extraction**

Total miRNA from cultured cells, serum, and surgically resected fresh cartilage tissues was isolated using the mirVana miRNA Isolation Kit (Ambion, Austin, TX, USA), according to the manufacturer’s instruction. The miRNA levels were assayed with Taqman probes and primer sets (Applied Biosystems, Foster City, CA, USA) in accordance with the manufacturer’s instructions. To perform absolute quantification in miRNA analyses, an HPLC-purified synthetic oligoribonucleotide standard identical in sequence to hsa-miR-18a was obtained commercially (Invitrogen, Carlsbad, CA, USA), which exact concentrations were measured by A260 measurements. Based on measured concentrations, standard curves with absolute copy counts were prepared by serial dilution.

**Western blotting**

Western blotting analysis was performed according to a standard method previously described (1), using anti-RUNX2 (Abcam, ab236639), anti-MMP13 (Abcam, ab84594), anti-COL10A1(Abcam, ab58632), anti-SMAD2 (Abcam, ab40855), anti-SMAD3 (Abcam, ab40854), anti-p-SMAD2 (Ser465/467) (Cell Signaling, Danvers, MA, USA, #18338), anti-p-SMAD3 (Ser423/425) (Cell Signaling, #9520), anti-SMAD2/3 (Cell Signaling, #8685), and anti-TGFβ1 (Abcam, ab215715) antibodies. Blotted membranes were stripped and re-blotted with an anti-β-actin (Sigma-Aldrich, A1978) antibody as a loading control.

**Plasmid construction and viral transduction**

To achieve overexpression of miR-18a, a DNA fragment containing the hsa-miR-18a precursor with 300 bp genomic sequences flanking each side was insert into the retroviral plasmid pMSCV-puro (Clontech, Palo Alto, CA, USA). The resultant plasmids were individually co-transfected with the pIK packaging plasmid in 293T cells by using a standard calcium phosphate transfection method as previously described (1). After 36 h, supernatants were collected and filtered to infect cells for 24 h in the presence of polybrene (2.5 μg/mL). Puromycin was used to select cells stably overexpressing miR-18a for 10 d. The open reading frames (ORFs) of SMAD2, SMAD3, and TGFβ1 generated by PCR amplification were separately cloned into the mammalian expression vector pcDNA3.1 (Invitrogen) and their 3’-UTRs were separately amplified into the downstream of the luciferase gene in a pGL3 control vector (Promega, Madison, WI, USA). The TGF-β- (p3TP-lux) reporter was obtained from Addgene Inc (Cambridge, MA, USA).

**Transfection**

Oligonucleotides including miR-18a mimics, inhibitor, antagomir, and corresponding NC controls were purchased from Ribobio (2). Transfection of plasmids or oligonucleotides was performed using Lipofectamine 3000 reagent (Invitrogen) for functional assays, luciferase reporter assays, or protein and RNA analyses.

**Luciferase reporter assay**

Cells were seeded in triplicates in 24-well plates and allowed to settle for 24 h. Indicated plasmids plus 1 ng pRL-TK renilla plasmid was transfected into the cells using the Lipofectamine 3000 reagent (Invitrogen). 48 h after transfection, Dual-Luciferase reporter assays were performed according to the manufacturer’s instruction (Promega) as previously described (1).

**RNA immunoprecipitation.**

Immunoprecipitation of miRNP with anti-Ago1 (Abcam) or anti-IgG (Sigma-Aldrich) was performed as previously described (2). In brief, cells were lysed in buffer containing 100mM KCl, 5mM MgCl2, 10mM HEPES (pH7.4), and 0.5% NP-40; and the immune complex captured by protein A agarose was washed in buffer containing 150mM KCl, 5mM MgCl_2_, 10mM HEPES (pH7.4), and 0.1% NP-40 for 6 times. RNA extraction was performed using RNAeasy Kit (Qiagen, Valencia, CA, USA).

**IHC analysis**

Paraffin sections (4 μm) were prepared, and IHC was performed with a Histostain-Plus kit (ZSGB-BIO, Beijing, China). The primary antibodies included anti-p-SMAD2/3, p65, and RUNX2 antibodies. A DAB Horseradish Peroxidase Color Development Kit (ZSGB-BIO) was used for detection. Immunostaining evaluations were performed independently by experimenters blinded to sample identity. The staining intensity was scored as follows: 0 (negative), 1 (weakly positive), 2 (moderately positive), and 3 (strongly positive). The percent of positivity was also scored according to 5 categories: 0 (<5%), 1 (5%−25%), 2 (25%−50%), 3 (50%−75%), and 4 (>75%) (3). Then, the value of the percent positivity score was multiplied by the staining intensity score to generate final expression scores, which ranged from 0 to 12.

**Statistical analysis**

All statistical analyses, except for microarray data, were performed using the SPSS 20.0 (IBM, Armonk, NY, USA) statistical software package. Patients were divided into two groups according to miR-18a expression levels divided by median expression value, and there were no significant differences in age and gender between the two groups. Comparisons between groups were performed using the Student’s t test. All error bars represent mean ± SD derived from three independent experiments. The *P* value less than 0.05 was considered statistically significant.

**Reference**

1. Liu L, et al. EGF-induced nuclear localization of SHCBP1 activates beta-catenin signaling and promotes cancer progression. *Oncogene.* 2019;38(5):747-64.

2. Yang Y, et al. MiR-503 targets PI3K p85 and IKK-beta and suppresses progression of non-small cell lung cancer. *Int J Cancer.* 2014;135(7):1531-42.

3. Liu L, et al. MTNR1B loss promotes chordoma recurrence by abrogating melatonin-mediated beta-catenin signaling repression. *J Pineal Res.* 2019;67(2):e12588.
